# Factors impacting the growth and nutritional status of cystic fibrosis patients younger than 10 years of age who did not undergo neonatal screening

**DOI:** 10.1016/j.rpped.2014.11.004

**Published:** 2015-03

**Authors:** Taís Daiene Russo Hortencio, Roberto José Negrão Nogueira, Fernando Augusto de Lima Marson, Gabriel Hessel, José Dirceu Ribeiro, Antônio Fernando Ribeiro

**Affiliations:** Universidade Estadual de Campinas, Campinas, SP, Brazil

**Keywords:** Cystic fibrosis, Child, Neonatal screening, Anthropometry, Nutritional status

## Abstract

**OBJECTIVE:**

The aim of this study was to evaluate by clinical and laboratory parameters how cystic fibrosis (CF) affects growth and nutritional status of children who were undergoing CF treatment but did not receive newborn screening.

**METHODS:**

A historical cohort study of 52 CF patients younger than 10 years of age were followed in a reference center in Campinas, Southeast Brazil. Anthropometric measurements were abstracted from medical records until March/2010, when neonatal screening program was implemented. Between September/2009 and March/2010, parental height of the 52 CF patients were also measured.

**RESULTS:**

Regarding nutritional status, four patients had Z-scores ≤-2 for height/age (H/A) and body mass index/age (BMI/A). The following variables were associated with improved H/A ratio: fewer hospitalizations, longer time from first appointment to diagnosis, longer time from birth to diagnosis and later onset of respiratory disease. Forced vital capacity [FVC(%)], forced expiratory flow between 25-75% of FVC [FEF_25-75_(%)], forced expiratory volume in the first second [FEV_1_(%)], gestational age, birth weight and early respiratory symptoms were associated with improved BMI/A.

**CONCLUSIONS:**

Greater number of hospitalizations, diagnosis delay and early onset of respiratory disease had a negative impact on growth. Lower spirometric values, lower gestational age, lower birth weight, and early onset of respiratory symptoms had negative impact on nutritional status. Malnutrition was observed in 7.7% of cases, but 23% of children had nutritional risk.

## Introduction

Cystic fibrosis (CF) is an autosomal recessive disease that is highly prevalent among caucasians. CF is characterized by the involvement of multiple organs, especially the pulmonary and gastrointestinal systems; by abnormally high levels of sweat chloride, and by increased incidences of male infertility and diabetes mellitus.^1^

Dietetic care is needed and individualized attention should be given to ensure adequate energy intake among CF patients. To maintain adequate nutritional status, CF children should ingest 110 to 150% of the daily caloric intake recommended for healthy children.^2^ Pancreatic insufficiency with chronic malabsorption, recurrent infections, chronic inflammation and energy expenditure, and insufficient nutritional intake are factors exacerbating malnutrition in CF patients. These factors lead to difficulties with weight maintenance and weight gain, and failure to thrive in infancy.

Regardless of the origin and reasons for high energy expenditure, the more clinically pertinent question is the influence of nutrition on the progression of lung disease, because lung function in CF patients is the main predictor of survival.^3^^,^ 4 Clinical studies indicate that nutritional status plays an important role in the progression of lung disease in CF and it is a survival advantage among patients with good nutritional status.^3,4^ These studies consistently support the strong influence of growth and nutritional status on CF-associated lung disease. But from birth, nutritional deficiency is determined primarily by pancreatic insufficiency and malabsorption, so subsequent aggressive nutritional support should facilitate proper growth and preserve lung function.

In Brazil, CF is associated with high morbidity and mortality. However, the survival of affected children in Brazil has increased substantially in the last 50 years due to an interdisciplinary approach to treatment, new medications and progress related to nutritional intervention and control. During the past 20 years, the benefit of early diagnosis on the nutritional status of CF patients has been established.^5^^,^ 6 However, failure-to-thrive diagnoses remain common despite early identification of CF.^7^^,^ 8

In this study, we use clinical and laboratory variables to assess how CF affects the growth and nutritional status of patients younger than 10 years who were undergoing CF treatment but did not receive newborn screening. 

## Method

A historical cohort study was designed to evaluate CF patients younger than 10 years from the CF Reference Center of the Hospital of Clinics of the University of Campinas (Unicamp, SP, Brazil). CF diagnoses were made when two sweat tests exceeded 60mEq/L for chloride and/or by genetic analyses with the identification of two *CFTR* mutations. Anthropometric measurements data weight and height were taken from medical records until March 2010, a time prior to the neonatal screening implementation, at the following times: birth, the first appointment with a medical specialist, at diagnosis and annually during each patient's birthday month. Between September 2009 and March 2010, parental heights also were measured. Patient weights were determined to the nearest 0.1kg using a digital electronic scale (Filizola^(r))^ with a 150kg capacity and 100 g accuracy. Heights were measured to the nearest 0.1cm using a wooden stadiometer. For children younger than 24 months, body length was measured with a horizontal child anthropometer. Clinical measurements were conducted in accordance with the Anthropometric Standardization Reference Manual.^9^ Z-scores were calculated for anthropometric indices including height/age (H/A) and body mass index/age (BMI/A) for all subjects. 

Z-score calculations were made using the programs WHO Anthro^10^ for children younger than five years and WHO Anthro PLUS^11^ for children aged five years and older. Nutritional status was classified according to anthropometric indices using the classification of the World Health Organization (WHO).^12^^,^ 13 Parental heights were measured to the nearest 0.1cm using a wooden stadiometer with an extension of 200cm displayed on a flat, vertical surface. Parental heights were transformed using the SISCRES program (Sistema de Análise do Crescimento - Growth Analysis System) into a distribution of Z-scores to obtain the mean and standard deviation based on CDC (Centers for Disease Control and Prevention) 2002 growth charts.^14^ Birth weights were classified according to WHO criteria.^15^

The nutritional treatment offered by this Reference Center is performed by a team composed of a dietitian and a physician specialized in nutrition. The state of São Paulo has offered free dietary supplements when prescribed by a doctor or dietitian to CF patients since 2003. At the first moment that weight and height progress is unsatisfactory, according to the current guidelines,^2^ oral supplements are usually advised. The enteral tube feeding is an option if the weight/height ratio falls below 85%, or even after trying oral energy supplements. Fat-soluble vitamins are always prescribed, and pancreatic enzymes are administered if there is evidence of pancreatic insufficiency. Vitamins and enzymes as well as drugs prescribed are offered by the State of São Paulo government.

Regarding genetic study, data were abstracted from medical records for the following CF-associated mutations: F508del, G542X, N1303K, G551D, R553X, and R1162X. Patients were classified as harboring two known CF alleles, one known allele, or no known allele. *CFTR* mutations were identified by polymerase chain reaction for the F508del mutation, and by the fragment-length polymorphism method for the G542X, R1162X, R553X, G551D and N1303K mutations. All *CFTR* mutations are included in classes I, II or III.

Patient medical records were reviewed for data regarding exclusive breastfeeding, presence of meconium ileus, onset of gastrointestinal and respiratory symptoms in months, number of hospitalizations per year, hepatopathy and pancreatic insufficiency.

Pulmonary function by spirometry test was performed in all CF patients with seven years of age, and the data was collected yearly until ten years of age. The fat balance and pulmonary microbiological data were collected, at the first appointment, at the reference center at diagnosis and annually during each patient's birthday month. Indicators were obtained a maximum of two months before or two months after the collection of anthropometric measurements. 

Pulmonary function was assessed by spirometry in children older than seven years. We used a model CPFS/D spirometer (MedGraphics, Medical Graphics Corporation^(r)^, MN, USA) and the spirometry software *BREEZE PF *version 3.8 B for Windows 95/98/NT (Medical Graphics Corp., MN, USA). During the test, the following parameters were evaluated: forced vital capacity (FVC), forced expiratory volume in the first second (FEV_1_), the Tiffenau Index (FEV_1_/FVC ratio) (TIFF) and forced expiratory flow between 25-75% of the FVC (FEF_25-75_%). The results followed the recommendations of the European Respiratory Society (ERS) and the American Thoracic Society (ATS),^16^ and all values were compared with the Polgar and Promadaht^17^ data and the absolute number was achieved for each patient included in the study. The values from the spirometry test were not adjusted by categories considering the severity. The absolute values of the data were considered, using numerical distribution to data analyses.^4^

The Van der Kamer method^18^ was used to assess fecal fat balance. Results were considered abnormal when fecal fat exceeded 2g/day in children aged 12 years or younger, or 5g/day in patients older than 12 years. The fecal fat balance was used to diagnose pancreatic insufficiency. 

Patients were also classified according to pulmonary colonization and infection by *Pseudomonas aeruginosa.* 19

The SAS System for Windows version 9.1.3 (SAS Institute Inc.; Cary, NC, USA) was used for statistical analysis. To examine the relationship between the evolution of weight/height and CF complications, generalized estimating equations (GEE)^20^ were applied to data from the same patient at different times. The variables were transformed into ranks due to the lack of a normal distribution. The significance level was set at *p*<0.05. The study was approved by the Ethics Committee of the School of Medical Sciences from State University of Campinas (#539/2008). The guardians of the patients gave written consent before the study began. 

## Results

The study population included 52 CF patients. Of these, 27/52 (51.9%) were female, and 49/52 (94.7%) were caucasian. The average patient age was 6 years and 9 months (±2,40). 

Regarding mutations in the *CFTR *gene, 26/52 (50%) carried two known CF-associated alleles, 21/52 (40.4%) carried one known allele, and 5/52 (9.6%) had no known alleles. Mutations were detected at the following frequency: F508del/F508del, 17 patients; F508del/G542X, five patients; F508del/N1303K, one patient; F508del/R1162X, two patients; F508del/R553X, one patient; F508del/no identified mutation, 19 patients; G542X/no identified mutation, one patient; R1162X/no identified mutation, one patient, and no identified mutation/no identified mutation, five patients. 

The median time between birth and diagnosis was 22 months (0-106.6 months). The median time of onset of gastrointestinal symptoms was 8 months (0-187.3 months), and the median time to respiratory symptoms was 14 months (0-223.1 months). The median time between the first visit in the service and the diagnosis was 2.4 months (0-66.8 months). 

Of the patients surveyed, 18.6% were born preterm, and 23.5% were born weighing <2.5kg. The mean birth weight was 2.9±0.6kg, the mean length was 47.5±2.6cm, and the mean duration of exclusive breastfeeding was 3.3±2.9 months. 

Meconium ileus, liver abnormalities and pancreatic insufficiency were present in 23%, 40% and 90.8% of patients, respectively. The patients had an average of 1.5 hospitalizations and a median of one hospitalization during the study period. Regarding lung infection/colonization, 64.3% of patients were chronically colonized by *P. aeruginos*,*a* and 69.2% had been submitted to spirometry. 

Patient's mothers and fathers had an average height of 1.62m and 1.73m, respectively. The parental target Z-score achieved by patients averaged 0.93. At birth, 25% of patients had Z-scores <-2 according to the H/A index, and 21% had Z-scores <-2 according to the BMI/A index. At their first appointment in the service, 38% of patients had Z-scores <-2 according to the H/A index. According to BMI/A, 40.4% had Z-scores <-2. At diagnosis, 32% of patients exhibited Z-scores <-2 according to the H/A, versus 7.7% in the current anthropometry (Table 1). Regarding BMI/A, 21% of patients had Z-scores <-2 at diagnosis, versus 7.7% in the current anthropometry (Table 2).

**Table 1:** Nutritional status of 52 CF patients aged less than 10 years according to gender and H/A index.

H/A Z-score	Number of patients	%	nM/F	%M/F
<–3 Z-Score	2	3.8	1/1	3.7/4.0
≥–3 Z-Score and <–2 Z-core	2	3.8	1/1	3.7/4.0
≥–2 Z-Score and <–1 Z-Score	16	30.8	9/7	33.3/28.0
≥–1 Z-Score and ≤+1 Z-Score	26	50.0.	12/14	44.4/56.0
>+1 Z-Score and ≤+2 Z-Score	5	9.6	4/1	14.8/4.0
>+2 Z-Score and ≤+3 Z-Score	1	1.9	0/1	0/4.0
>+3 Z-Score	0	0.	0/0	0/0

n, number of patients; %, percentage; M, male; F, female; H/A, Height/Age.

**Table 2:** Nutritional status of 52 CF patients aged less than 10 years according to gender and the BMI/A index.

BMI/A Z-score	Number of patients	%	nM/F	%M/F
<–3 Z-Score	2	3.8	0/2	0/8
≥–3 Z-Score and <–2 Z-core	2	3.8	2/0	8/0
≥–2 Z-Score and <–1 Z-Score	12	23.1	6/6	22/24
≥–1 Z-Score and ≤+1 Z-Score	30	57.7	14/16	52/64
>+1 Z-Score and ≤+2 Z-Score	5	9.6	5/0	18.5/0
>+2 Z-Score and ≤+3 Z-Score	1	1.9	0/1	0/4
>+3 Z-Score	0	0.	0/0	0/0

n, number of patients; %, percentage; M, male; F, female; BMI/A, body mass index/age.

Table 3 shows the results for the estimation obtained by GEE for the H/A index. Better correlated H/A values were related to fewer hospitalizations, greater time between the first appointment and diagnosis, greater time between birth and diagnosis and later onset of respiratory symptoms.

**Table 3:** Relationship between clinical and laboratory CF variables and H/A index using generalized linear models with GEE (Generalized Estimating Equations).

Variables	Estimate	Standard error	95%CI		*p *value
*Pseudomonas aeruginosa*					
No	0.203	0.123	—0.038	0.444	0.099
Yes	0	0	0	0	—
Pancreatic Insufficiency					
No	0.169	0.137	—0.099	—0.437	0.217
Yes	0	0	0	0	—
Hepatopathy					
No	0.066	—0.123	—0.176	—0.308	0.593
Yes	0	0	0	0	—
Spirometry					
FVC (%)	—0.001	0.003	—0.007	0.005	0.788
FEV^1^ (%)	—0.001	0.004	—0.009	0.006	0.749
FVC/FEV^1^ (%)	—0.007	—0.007	—0.017	0.004	0.204
FEF^25-75^ (%)	—0.002	—0.001	—0.004	—0.004	0.938
Gestational age					
Term	0.177	0.128	—0.073	—0.428	0.166
Preterm	0	0	0	0	—
Birth weight					
≥2.5kg	0.704	1.054	1.367	2.769	0.504
<2.5kg	0	0	0	0	—
Meconium ileus					
Yes	—0.091	0.122	—0.329	0.147	0.452
No	0	0	0	0	—
Period of exclusive breastfeeding					
—	0.028	0.017	0.005	0.061	0.094
Number of hospitalizations					
—	—0.101	0.022	—0.144	–0.058	<0.001
*Time from 1* ^st^ * appointment to diagnosis*					
­—	0.004	0.001	0.001	0.007	0.004
Time between birth and diagnosis					
—	0.004	0.001	0.002	0.006	<0.001
Known alleles					
1 known allele	—0.023	0.131	—0.280	0.234	0.861
2 known alleles	—0.044	0.146	—0.330	0.242	0.764
No known alleles	0	0	0	0	—
Onset of gastrointestinal symptoms					
—	0.002	0.001	—0.001	0.004	0.161
Onset of respiratory symptoms					
—	0.004	0.002	0.001	0.007	0.010

H/A, Height/age; FVC (%), forced vital capacity; FEV1 (%), forced expiratory volume in the first second; FEF_25-75_ (%), forced expiratory flow between 25-75% of FVC; CI, confidence interval.

Table 4 summarizes the results of the GEE analysis for the BMI/A index. Better correlated BMI/A values were associated with higher FVC, FEV_1_, FEF_25-75_% values; longer gestational age; greater birth weight, and later onset of respiratory symptoms.

**Table 4:** Relationship between clinical and laboratory CF variables and BMI/A index using generalized linear models with GEE (Generalized Estimating Equations).

Variables	Estimate	Standard error	95%CI		*p* value
*Pseudomonas aeruginosa* **					
No	—0.021	0.082	—0.182	0.140	0.799
Yes	0	0	0	0	—
Pancreatic Insufficiency					
No	—0.056	0.100	—0.251	0.139	0.573
Yes	0	0	0	0	—
Hepatopathy					
No	—0.071	—0.071	—0.091	—0.240	0.436
Yes	0	0	0	0	—
Spirometry					
FVC (%)	0.008	0.003	0.003	0.013	0.002
FEV^1^ (%)	0.009	0.003	0.004	0.014	0.001
FVC/FEV^1^ (%)	—0.001	0.005	—0.010	0.008	0.824
FEF^25-75^ (%)	0.004	0.002	0.001	0.007	0.008
Gestational age					
Term	0.378	0.123	0.136	0.619	0.002
Preterm	0	0	0	0	—
Birth weight					
≥2.5kg	0.479	0.115	0.255	0.703	<0.001
<2.5kg	0	0	0	0	—
Meconium ileus					
Yes	—0.118	0.111	—0.335	0.098	0.285
No	0	0	0	0	—
Period of exclusive breastfeeding					
—	0.017	0.016	—0.014	0.047	0.281
Number of hospitalizations					
—	—0.022	0.027	—0.076	0.032	0.422
*Time from 1* ^st^ * appointment to diagnosis*					
—	0.002	0.002	—0.002	0.005	0.294
Time between birth and diagnosis					
—	0.002	0.001	—0.001	0.004	0.065
Known alleles					
1 known allele	0.065	0.145	—0.224	0.355	0.657
2 known alleles	0.004	0.153	—0.254	0.346	0.763
No known allele	0	0	0	0	—
Onset of gastrointestinal symptoms					
—	0.002	0.001	0	0.003	0.054
Onset of respiratory symptoms					
—	0.002	0.001	0.001	0.004	0.014

BMI/A, body mass index/age; FVC (%), forced vital capacity; FEV_1_ (%), forced expiratory volume in the first second; FEF_25-75_ (%), forced expiratory flow between 25-75% of FVC; CI, confidence interval.

## Discussion

The age at diagnosis is an important factor for the nutritional status of CF patients. Early diagnosis facilitates special attention to nutritional status, growth curve monitoring and detection of pathogen colonization in upper airways, which is intimately related with a worse prognosis. On this historical cohort, we discuss about how CF manifestations can affect the growth and the nutritional status of affected children.

Patients diagnosed early are closer to normal nutritional status at diagnosis and during the following 10 years.^5^ In this study, the median time between birth and diagnosis was 22 months, and the median time between the first appointment and diagnosis was 2.4 months. 

North American and European data report median ages at CF diagnosis of six months and five months, respectively.^21^^,^ 22 Late CF diagnosis occurs in developed countries among patients with distinct genotypic expression, milder pulmonary disease and absent gastrointestinal symptoms.^23^ In a developing country like Brazil, aside from the aforementioned reasons, delayed diagnosis appears to result from late forwarding of patients to a reference center, because the disease is not immediately recognized. 

In our study, longer times between birth and diagnosis and between the first appointment and diagnosis were associated with better correlated H/A; moreover, delayed respiratory symptom onset was associated with better H/A and BMI/A ratios. These associations are probably due to the presence of patients with mild lung disease and no suggestive symptoms of CF, and consequently, a smaller impact of the disease on growth and nutritional status, as it is known that improvement in pulmonary function is associated with increased BMI during childhood.^24^

There was improved nutritional status with increased FVC(%), FEV_1_(%) and FEF_25-75_% values, as analyzed by the BMI/A index. 

According to the literature, lung health depends on the patient's initial nutritional status. Patients who recover from poor nutritional status up to two years after the diagnosis show better pulmonary function and less coughing at six years of age, emphasizing the need for comprehensive and aggressive treatment, implemented as soon as possible after diagnosis.^25^

Weight and height changes have been recognized as one of the prognostic factors of severity of the disease and shorter survival in CF patients. A limitation of our study is that spirometry was done on patients older than seven years old, not including the entire CF patient population. 

The incidence of low height and weight decreased during treatment in this study. At the first appointment in the service, 40.4% of patients had Z-scores <-2 according to the BMI/A index, versus 7.7% of patients assessed before the neonatal screening implementation. As for the H/A index, 38% of patients had Z-scores <-2, versus 7.7%. 

The high percentage of CF patients with meconium ileus (23%) could justify the high percentage of patients who were malnourished in their first years of life. Patients with meconium ileus treated surgically in the first days of life undergo aggravating effects that can impair their nutritional status, and the surgical stress may difficult feeding postoperatively, during an important phase of growth. But these patients had a good nutritional recovery without compromising their nutritional status and growth. Due to improvements in neonatal surgical techniques, better postoperative care and nutritional support, the nutritional status and survival of CF patients with meconium ileus appear to be similar to those of CF patients without meconium ileus. The incidence of meconium ileus reported by Alvarez et al, at the same CF Reference Center, was 5.8%.^23^ The hypothesis associated with the low incidence of meconium ileus in the last study was that CF patients would die in the first year of life, before the diagnosis was performed.^23^ In the present study, we detected meconium ileus in 23% of patients, a value close to the 15-20% mentioned in the international literature.^26^ Currently, with the neonatal screening, the management of meconium ileus may be more effective in patients with CF.

Height is an inherited growth trait deeply influenced by nutrition and diseases. Parents of CF patients had higher average heights compared with the average population. Average heights were 1.73m for fathers and 1.62m for mothers in the present study. 

According to the Brazilian Institute of Geography and Statistics (IBGE), the average height of Brazilian men was 1.70m, and of Brazilian women was 1.58m in 2009.^27^ The genetic contributions of parents to the height of their offspring are influenced by nutrition and diseases, especially chronic diseases such as CF. The height of the studied patients, considering the parental target, was adequate. This is incongruent with data obtained by other studies that reported a decreased average height among CF children compared with average parental height.^28^ Thus, despite late diagnoses (median of 22 months in this study versus six months in developed countries), the final heights of the children in this study were not impaired, which probably reflects the appropriate multidisciplinary care offered to patients. These data indicate that the nutritional status of our patients may have been similar to those obtained in major centers in developed countries.

A child with a Z-score ranking in the range ≥-2 and <-1, suggesting that the child is approaching categories of low weight or height for their age, deserves the attention of health professionals and caregivers. A child with this range should be considered as risk group for nutritional monitoring. In this study, 30.8% of patients according to the H/A index and 23% of patients according to the BMI/A index were within this monitoring range of nutritional risk. It is noteworthy that most patients were of adequate height and weight, according to the studied indices, but weight changes in CF are very frequent, requiring special attention to these events.

CF children have pancreatic insufficiency at birth. Low birth weight is present even in the absence of prematurity, as changes in fetal growth due to the malfunctioning exocrine pancreas reduce intrauterine nutrition and precipitate lower birth weights.^29^ In our study, 23.5% of patients had low birth weight, and 18.6% were preterm infants. These values exceed those reported by Festini et al, who evaluated the neonatal characteristics of children with CF in Italy.^30^ They showed that the weights of newborns with CF are in average 246g lower than those of the non-CF population, and that CF children have a higher risk of being born preterm, small for gestational age and with a low birth weight than children without CF.^30^

Patients who are less often hospitalized have better H/A relationship. Among CF patients, the increased need for energy intake is associated with increased caloric demand due to repetitive airway infections and inflammation, decreased food intake during the episodes of acute respiratory disease and decreased fat absorption. This energy deficit causes or worsens malnutrition and is common in hospitalized patients. It leads to growth retardation, decreased muscle strength, fatigue, recurrent respiratory infections and diminished lung function, thereby decreasing survival in CF patients.

Despite the high prevalence of preterm birth, low birth weight and *P. aeruginosa* colonization, the studied patients exhibited good nutritional status and growth during childhood. Probably, the treatment offered in our reference center is associated to this outcome. The Reference Center offers appointments with health professionals of four medical specialties: pulmonology, gastroenterology, physiotherapy and nutrition, and the state of São Paulo, in Brazil, offers free dietary supplements when prescribed by a doctor or dietitian to CF patients since 2003. This policy has ensured better adherence to treatment and better nutrition. Furthermore, a non-governmental organization formed by parents, called "Fibrocis", works in tandem, offering home visits made by social workers and financial assistance for patient transportation to appointments.

The present study is unique because it is the first to evaluate how several CF manifestations affect the growth and nutritional status of children who were undergoing CF treatment but were not diagnosed at birth by newborn screening in Brazil. However, there are several limitations. Although some results are based on a form filled by doctors responsible for these patients, some tests are not routinely performed due to cost constraints and government funding restrictions. Another limitation is that data collection was performed at a single major center in Brazil, and our results may not reflect the nutritional status and growth of all Brazilian children with CF. 

In conclusion, several CF manifestations influenced growth and nutritional status during treatment, but these patients exhibited good nutritional status, with a low rate of malnutrition and adequate growth. However, many of them were in the range of nutritional risk, requiring special attention to weight loss and proper growth. Appropriate nutritional management detemines outcomes. 

It is crucial that dietitians, pediatricians and other professionals involved in the care of CF patients recognize the growth process of patients since the first appointment to allow for early intervention upon diagnosis, before significant malnutrition occurs. Attention given at childhood can impact on nutritional status, growth, pulmonary health and treatment adherence, and it can enhance the survival and quality-of-life of patients with CF. 
