# Supplementary material for: Factors impacting the growth and nutritional status of cystic fibrosis patients younger than 10 years of age who did not undergo neonatal screening
Source: Rev Paul Pediatr. 2015 Mar;33(1):3–11. doi: 10.1016/j.rpped.2014.11.004 (PMC4436950; doi:10.1016/j.rpped.2014.11.004)
Supplement: Translation

# Translation: Fatores que afetam o crescimento e estado nutricional de pacientes com fibrose cística com idade inferior a 10 anos e que não foram submetidos à triagem neonatal

**Authors:** Taís Daiene Russo Hortencio, Roberto José Negrão Nogueira, Fernando Augusto de Lima Marson, Gabriel Hessel, José Dirceu Ribeiro, Antônio Fernando Ribeiro

**Affiliations:** Universidade Estadual de Campinas (Unicamp), Campinas, SP, Brasil

**Keywords:** Fibrose cística/complicações, Criança, Triagem neonatal, Antropometria, Estado nutricional

## Abstract

**OBJETIVO:**

Avaliar por meio de parâmetros clínicos e laboratoriais como a fibrose cística (FC) afeta o crescimento e estado nutricional de crianças submetidas ao tratamento de FC que não foram submetidas à triagem neonatal.

**MÉTODOS:**

Uma coorte histórica com 52 pacientes com FC menores de 10 anos foi acompanhada em um centro de referência em Campinas, Sudeste do Brasil. Peso e altura foram coletados de prontuários médicos até março de 2010, quando a triagem neonatal foi implementada. Entre setembro de 2009 a março de 2010 a altura dos pais foi medida.

**RESULTADOS:**

Quatro pacientes tiveram escores Z ≤ -2 para altura/idade (A/I) e índice de massa corporal/idade (IMC/A). As seguintes variáveis foram associadas com melhor razão A/I: menor número de hospitalizações, maior tempo entre a primeira consulta e o diagnóstico, maior tempo entre o nascimento e o diagnóstico e início tardio da doença respiratória. Capacidade vital forçada [CVF(%)], fluxo expiratório forçado entre 25-75% da CVF [FEF_25-75_(%)], volume expiratório forçado no primeiro segundo [VEF_1_(%)], idade gestacional, peso ao nascer e início dos sintomas respiratórios foram associados com melhor IMC/I.

**CONCLUSÕES:**

Maior número de hospitalizações, retardo no diagnóstico e início precoce da doença respiratória tiveram impacto negativo no crescimento. Menores valores espirométricos, menor idade gestacional, menor peso ao nascer e o início precoce dos sintomas respiratórios tiveram impacto negativo no estado nutricional. A desnutrição foi observada em 7,7% dos casos, mas 23% das crianças apresentaram risco nutricional.

## Introdução

A fibrose cística (FC) é uma doença autossômica recessiva altamente prevalente entre brancos. A FC é caracterizada pelo envolvimento de múltiplos órgãos, especialmente os sistemas gastrointestinais e pulmonares; por níveis anormalmente elevados de cloreto no suor; e pelo aumento da incidência de infertilidade masculina e *diabetes mellitus*. 1

O cuidado dietético é necessário e deve-se dar uma atenção individualizada para garantir a ingestão adequada de energia entre os pacientes com FC. Para manter o estado nutricional adequado, crianças com FC devem ingerir de 110 a 150% da ingestão calórica diária recomendada para crianças saudáveis.^2^ A insuficiência pancreática com má absorção crônica, infecções recorrentes, inflamação crônica e gasto energético e ingestão nutricional insuficiente são fatores agravantes da desnutrição em pacientes com FC. Esses fatores levam à dificuldades com a manutenção e o ganho do peso e o déficit de crescimento na infância.

Independentemente da origem e dos motivos para o alto gasto energético, a questão mais pertinente clinicamente é a influência da nutrição sobre a progressão da doença pulmonar, porque a função pulmonar em pacientes com FC é o principal preditor de sobrevida.^3^ ^and^ 4 Os estudos clínicos indicam que o estado nutricional desempenha um papel importante na progressão da doença pulmonar na FC e é uma vantagem de sobrevivência entre os pacientes com bom estado nutricional.^3^ ^and^ 4 Esses estudos apoiam de forma consistente a forte influência do crescimento e estado nutricional na doença pulmonar associada à FC. Mas, desde o nascimento, a deficiência nutricional é determinada principalmente pela insuficiência pancreática e má absorção, portanto o suporte nutricional agressivo subsequente deve facilitar o crescimento adequado e preservar a função pulmonar.

No Brasil, a FC é associada a alta morbidade e mortalidade. No entanto, a sobrevivência das crianças afetadas no Brasil tem aumentado substancialmente nos últimos 50 anos, devido a uma abordagem interdisciplinar para o tratamento, novos medicamentos e avanços relacionados com a intervenção e o controle nutricional. Durante os últimos 20 anos foi estabelecido o benefício do diagnóstico precoce no estado nutricional de pacientes com FC.^5^ ^and^ 6 No entanto, diagnósticos de falha de crescimento continuam a ser comuns, apesar da identificação precoce da FC.^7^ ^and^ 8

Neste estudo, usamos variáveis clínicas e laboratoriais para avaliar como a FC afeta o crescimento e o estado nutricional de pacientes com menos de 10 anos, submetidos a tratamento para FC, mas que não foram submetidos à triagem neonatal.

## Métodos

Um estudo de coorte histórica foi elaborado para avaliar pacientes com FC com idade inferior a 10 anos do Centro de Referência de FC do Hospital das Clínicas da Universidade de Campinas (Unicamp, SP, Brasil). Os diagnósticos de FC foram feitos quando dois testes de suor excederam 60 mEq/L de cloreto e/ou por meio de análises genéticas com a identificação de duas mutações *CFTR*. As medidas antropométricas de peso e altura foram obtidas de prontuários médicos até março de 2010, período anterior à implantação da triagem neonatal, nos seguintes tempos: nascimento, primeira consulta com um médico especialista, no momento do diagnóstico e anualmente no mês de aniversário de cada paciente. Entre setembro de 2009 e março de 2010, a altura dos pais também foi medida. Os pesos dos pacientes foram determinados com uma precisão de 0,1 kg com uma balança digital eletrônica (Filizola(r), SP, Brasil) com capacidade para 150 kg e precisão de 100 g. A estatura foi determinada com precisão de 0,1 cm, com um estadiômetro de madeira. Para as crianças com menos de 24 meses, o comprimento do corpo foi medido com um antropômetro infantil horizontal. Medidas clínicas foram feitas de acordo com o Manual de Referência Antropométrica. 9 Os escores Z foram calculados para os índices antropométricos, incluindo altura/idade (A/I) e índice de massa corporal/idade (IMC/I) para todos os indivíduos.

Os cálculos dos escores Z foram feitos com os programas WHO Anthro^10^ para crianças com menos de cinco anos e WHO Anthro PLUS^11^ para as crianças de cinco anos ou mais. O estado nutricional foi classificado de acordo com índices antropométricos da Organização Mundial da Saúde (OMS).^12^ ^and^ 13 As alturas dos pais foram medidas com precisão de 0,1 cm, com um estadiômetro de madeira com uma extensão de 200 centímetros colocado sobre uma superfície plana e vertical. As alturas dos pais foram transformadas com o programa Siscres (Sistema de Análise do Crescimento) em uma distribuição de escores-Z para obter a média e o desvio-padrão com base nas tabelas de crescimento de 2002 do CDC (Centers for Disease Control and Prevention).^14^ O peso ao nascer foi classificado segundo os critérios da OMS.^15^

O tratamento nutricional oferecido por esse Centro de Referência é feito por uma equipe composta por um nutricionista e um médico especializado em nutrição. Desde 2003 o Estado de São Paulo oferece suplementos dietéticos livres de custo quando prescritos por um médico ou nutricionista para pacientes com FC. No primeiro momento em que o progresso de peso e altura não é satisfatório, de acordo com a diretriz atual,^2^ suplementos orais são geralmente sugeridos. A alimentação por sonda enteral é uma opção se a razão peso/altura cair abaixo de 85% ou mesmo após a tentativa de uso de suplementos energéticos orais. Vitaminas lipossolúveis são sempre prescritas e enzimas pancreáticas são administradas se houver evidência de insuficiência pancreática.

Em relação ao estudo genético, os dados foram extraídos dos registros médicos para as seguintes mutações associadas com a FC: F508del, G542X, N1303 K, G551D, R553X e R1162X. Os pacientes foram classificados como portadores de dois alelos conhecidos da FC, de um alelo conhecido ou sem alelo conhecido. As mutações *CFTR* foram identificadas pela reação de polimerase em cadeia para a mutação F508del e pelo método do polimorfismo de comprimento de fragmento para as mutações G542X, R1162X, R553X, G551D e N1303 K. Todas as mutações CFTR estão incluídas nas classes I, II ou III.

Prontuários médicos foram revisados para obter dados a respeito de amamentação exclusiva, presença de íleo meconial, início dos sintomas gastrointestinais e respiratórios em meses, número de hospitalizações por ano, hepatopatia e insuficiência pancreática.

A função pulmonar por espirometria foi avaliada em todos os pacientes com FC e idade de sete anos e os dados foram coletados anualmente até os 10 anos. Os dados do balanço de gordura e microbiológico pulmonar foram coletados na primeira consulta no centro de referência, no diagnóstico e anualmente durante o mês de aniversário de cada paciente. Os indicadores foram obtidos, no máximo, dois meses antes ou dois meses após a coleta das medidas antropométricas.

Foi usado um espirômetro modelo CPFS/D (MedGraphics, Medical Graphics Corporation(r), MN, USA) e o *software* de espirometria Breeze PF, versão 3.8 B para Windows 95/98/NT (Medical Graphics Corp., MN, EUA). Durante o teste, foram avaliados os seguintes parâmetros: capacidade vital forçada (CVF), volume expiratório forçado no primeiro segundo (FEV_1_), Índice de Tiffenau (razão FEV1/CVF) (TIFF) e fluxo expiratório forçado entre 25-75% da CVF (FEF_25-75%_). Os resultados seguiram as recomendações da European Respiratory Society (ERS) e American Thoracic Society (ATS), 16 todos os valores foram comparados com os dados de Polgar e Promadaht 17 e o número absoluto foi obtido para cada paciente incluído no estudo. Os valores do teste de espirometria não foram ajustados por categorias considerando a gravidade. Foram considerados os valores absolutos dos dados, com o uso da a distribuição numérica para análise. 4

O método de Van der Kamer^18^ foi usado para avaliar o balanço de gordura fecal. Os resultados foram considerados anormais quando a gordura fecal ultrapassou 2 g/dia em crianças de até 12 anos ou menos ou 5 g/dia em pacientes com mais de 12 anos. O balanço de gordura nas fezes foi usado para diagnosticar a insuficiência pancreática.

Os pacientes também foram classificados de acordo com a colonização e infecção pulmonar por *Pseudomonas aeruginosa*. 19

O sistema SAS para Windows versão 9.1.3 (SAS Institute Inc.; Cary, NC, EUA) foi usado para análise estatística. Para analisar a relação entre a evolução do peso/altura e complicações da FC, as equações de estimação generalizadas (GEE)^20^ foram aplicadas aos dados do mesmo paciente em momentos diferentes. As variáveis foram transformadas em categorias devido à falta de uma distribuição normal. O nível de significância foi estabelecido como p < 0,05. O estudo foi aprovado pelo Comitê de Ética da Faculdade de Ciências Médicas da Universidade Estadual de Campinas (n° 539/2008). Os responsáveis pelos pacientes deram consentimento por escrito antes do início do estudo.

## Resultados

A população do estudo incluiu 52 pacientes com FC. Desses, 27/52 (51,9%) eram do sexo feminino e 49/52 (94,7%) eram brancos. A idade média dos pacientes era de seis anos e nove meses (± 2,40).

Em relação às mutações no gene *CFTR*, 26/52 (50%) eram portadores de dois alelos conhecidos associados à FC, 21/52 (40,4%) tinham um alelo conhecido e 5/52 (9,6%) não apresentavam alelos conhecidos. As mutações foram detectadas nas seguintes frequências: F508del/F508del, 17 pacientes; F508del/G542X, cinco pacientes; F508del/N1303 K, um paciente; F508del/R1162X, dois pacientes; F508del/R553X, um paciente; F508del/mutação não identificada, 19 pacientes; G542X/mutação não identificada, um paciente; R1162X/mutação não identificada, um paciente e mutação não identificada/mutação não identificada, cinco pacientes.

O tempo mediano entre o nascimento e o diagnóstico foi de 22 meses (0-106,6). O tempo mediano para o aparecimento dos primeiros sintomas gastrointestinais foi de oito meses (0-187,3) e o tempo mediano para o aparecimento dos sintomas respiratórios foi de 14 meses (0-223,1). O tempo mediano entre a primeira consulta no serviço e o diagnóstico foi de 2,4 meses (0-66,8).

Dos pacientes estudados, 18,6% nasceram prematuros e 23,5% nasceram com peso <2,5 kg. O peso médio ao nascer foi de 2,9±0,6 kg, a altura média foi de 47,5±2,6 cm e a duração média do aleitamento materno exclusivo foi de 3,3±2,9 meses.

Íleo meconial, anormalidades do fígado e insuficiência pancreática estavam presentes em 23%, 40% e 90,8% dos pacientes, respectivamente. Os pacientes tiveram uma média de 1,5 hospitalização e mediana de uma hospitalização durante o período de estudo. Em relação à infecção/colonização pulmonar, 64,3% dos pacientes eram colonizados cronicamente pela *P. aeruginosa* e 69,2% haviam sido submetidos à espirometria.

As mães e pais dos pacientes tinham uma altura média de 1,62 m e 1,73 m, respectivamente. O escore Z de alvo parental alcançado pelos pacientes foi em média 0,93. Ao nascer, 25% dos pacientes tiveram escores Z <-2 de acordo com o índice A/I e 21% tinham escores -Z <-2 de acordo com o índice IMC/I. Na sua primeira consulta no serviço, 38% dos pacientes tinham escores Z <-2 de acordo com o índice A/I. De acordo com o IMC/I, 40,4% tiveram escores Z <-2. No momento do diagnóstico, 32% dos pacientes apresentaram escore Z <-2 de acordo com o índice A/I *versus* 7,7% na antropometria atual (tabela 1). Em relação ao IMC/I, 21% dos pacientes tiveram escores -Z <-2 no diagnóstico *versus* 7,7% na antropometria atual (tabela 2).

**Tabela 1:** Estado nutricional de 52 pacientes com FC e idade menor de 10 anos de acordo com o sexo e o índice A/I

A/I Escore Z	Número de pacientes	%	n M/F	% M/F
<-3 Escore Z	2	3,8	1/1	3,7/4
≥-3 Escore Z and <-2 Z-core	2	3,8	1/1	3,7/4
≥-2 Escore Z and <-1 Escore Z	16	30,8	9/7	33,3/28
≥-1 Escore Z and ≤+1 Escore Z	26	50.0	12/14	44,4/56
>+1 Escore Z and ≤+2 Escore Z	5	9,6	4/1	14,8/4
>+2 Escore Z and ≤+3 Escore Z	1	1,9	0/1	0/4
>+3 Escore Z	0	0	0/0	0/0

n, número de pacientes; %, porcentagem; M, masculino; F, feminino; A/I, altura/idade.

**Tabela 2:** Estado nutricional de 52 pacientes com FC e idade menor de 10 anos de acordo com o sexo e o índice IMC/I

IMC/I Escore Z	Número de pacientes	%	n M/F	% M/F
<-3 Escore Z	2	3,8	0/2	0/8,0
≥-3 Escore Z and <-2 Escore Z	2	3,8	2/0	8,0/0
≥-2 Escore Z and <-1 Escore Z	12	23,1	6/6	22,2/24,0
≥-1 Escore Z and ≤+1 Escore Z	30	57,7	14/16	51,9/64,0
>+1 Escore Z and ≤+2 Escore Z	5	9,6	5/0	18,5/0
>+2 Escore Z and ≤+3 Escore Z	1	1,9	0/1	0/4,0
>+3 Escore Z	0	0	0/0	0/0

n, o número de pacientes; %, porcentagem; M, masculino; F, feminino; IMC/I, índice de massa corporal/idade.

A tabela 3 mostra os resultados para a estimativa obtida por GEE para o índice A/I. Os valores de A/I mais bem correlacionados estavam associados com menos hospitalizações, maior tempo entre a primeira consulta e o diagnóstico, maior tempo entre o nascimento e o diagnóstico e sintomas respiratórios de início retardado.

**Tabela 3:** Associação entre variáveis clínicas e laboratoriais da FC e o índice A/I com ou uso de modelos lineares generalizados com GEE (Equações de Estimação Generalizadas)

Variáveis	Estimativa	Erro padrão	IC95%		Valor *p*
*Pseudomonas aeruginosa*					
Não	0,203	0,123	–0,038	0,444	0,099
Sim	0	0	0	0	-

*Insuficiência pancreática*					
Não	0,169	0,137	–0,099	–0,437	0,217
Sim	0	0	0	0	-

*Hepatopatia*					
Não	0,066	–0,123	–0,176	–0,308	0,593
Sim	0	0	0	0	-

*Espirometria*					
CVF(%)	–0,001	0,003	–0,007	0,005	0,788
FEV_1_(%)	–0,001	0,004	–0,009	0,006	0,749
CVF/FEV_1_(%)	–0,007	–0,007	–0,017	0,004	0,204
FEF_25-75_(%)	–0,002	–0,001	–0,004	–0,004	0,938

*Idade gestacional*					
Termo	0,177	0,128	–0,073	–0,428	0,166
Prematuro	0	0	0	0	-

*Peso ao nascer*					
≥ 2,5 kg	0,704	1,054	1,367	2,769	0,504
< 2,5 kg	0	0	0	0	-

*Íleo meconial*					
Sim	–0,091	0,122	–0,329	0,147	0,452
Não	0	0	0	0	-

*Período de amamentação exclusiva*					
-	0,028	0,017	0,005	0,061	0,094

*Número de hospitalizações*					
-	–0,101	0,022	–0,144	-0,058	<0,001

*1ª consulta - diagnóstico*					
-	0,004	0,001	0,001	0,007	0,004

*Nascimento - diagnóstico*					
-	0,004	0,001	0,002	0,006	<0,001

*Alelos conhecidos*					
1 alelo conhecido	–0,023	0,131	–0,280	0,234	0,861
2 alelos conhecidoss	–0,044	0,146	–0,330	0,242	0,764
Alelos não conhecidos	0	0	0	0	-

*Início dos sintomas gastrintestinais*					
-	0,002	0,001	–0,001	0,004	0,161

*Início dos sintomas respiratórios*					
-	0,004	0,002	0,001	0,007	0,010

A/I, altura/idade; CVF (%), capacidade vital forçada; FEV1 (%), volume expiratório forçado no primeiro segundo; FEF_25-75_ (%), fluxo expiratório forçado entre 25-75% da CVF; IC, intervalo de confiança; D, intervalo de tempo.

A tabela 4 resume os resultados da análise por GEE para o índice IMC/I. Os valores de IMC/I mais bem correlacionados estavam associados com valores mais elevados de CVF, VEF1, FEF_25-75%_; idade gestacional mais longa; maior peso no nascimento e sintomas respiratórios de início retardado.

**Tabela 4:** Associação entre variáveis clínicas e laboratoriais da FC e o índice IMC/I com o uso de modelos lineares generalizados com GEE (Equações de Estimação Generalizadas)

Variáveis	Estimativa	Erro padrão	IC95%		*p* valor
*Pseudomonas aeruginosa*					
Não	–0,021	0,082	–0,182	0,140	0,799
Sim	0	0	0	0	**-**

*Insuficiência pancreática*					
Não	–0,056	0,100	–0,251	0,139	0,573
Sim	0	0	0	0	**-**

*Hepatopatia*					
Não	–0,071	–0,071	–0,091	–0,240	0,436
Sim	0	0	0	0	**-**

*Espirometria*					
CVF(%)	0,008	0,003	0,003	0,013	0,002
FEV_1_(%)	0,009	0,003	0,004	0,014	0,001
CVF/FEV_1_(%)	–0,001	0,005	–0,010	0,008	0,824
FEF_25-75_(%)	0,004	0,002	0,001	0,007	0,008

*Idade gestacional*					
Termo	0,378	0,123	0,136	0,619	0,002
Pré-termo	0	0	0	0	**-**

*Peso ao nascer*					
≥ 2,5 kg	0,479	0,115	0,255	0,703	<0,001
< 2,5 kg	0	0	0	0	**-**

*Íleo meconial*					
Sim	–0,118	0,111	–0,335	0,098	0,285
Não	0	0	0	0	**-**

*Período de amamentação exclusiva*					
-	0,017	0,016	–0,014	0,047	0,281

*Número de hospitalizações*					
-	–0,022	0,027	–0,076	0,032	0,422

*1ª consulta - diagnóstico*					
-	0,002	0,002	–0,002	0,005	0,294

*Nascimento - diagnóstico*					
-	0,002	0,001	–0,001	0,004	0,065

*Alelos conhecidos*					
1 alelo conhecido	0,065	0,145	–0,224	0,355	0,657
2 alelos conhecidos	0,004	0,153	–0,254	0,346	0,763
Nenhum alelo conhecido	0	0	0	0	-

*Início dos sintomas gastrintestinais*					
-	0,002	0,001	0	0,003	0,054

*Início dos sintomas respiratórios*					
-	0,002	0,001	0,001	0,004	0,014

IMC/I, índice de massa corporal/idade; CVF (%), capacidade vital forçada; FEV1 (%), volume expiratório forçado no primeiro segundo; FEF25-75 (%), fluxo expiratório forçado entre 25-75% da CVF; IC, intervalo de confiança.

## Discussão

A idade em que é feito o diagnóstico é um fator importante para o estado nutricional de pacientes com FC. O diagnóstico precoce facilita uma atenção especial ao estado nutricional, ao acompanhamento da curva de crescimento e à detecção de colonização de patógenos nas vias aéreas superiores, os quais estão intimamente relacionados a um pior prognóstico. Nessa coorte histórica, discutimos como as manifestações de FC podem afetar o crescimento e o estado nutricional dessas crianças.

Pacientes diagnosticados precocemente estão mais próximos do estado nutricional normal no momento do diagnóstico e nos 10 anos seguintes.^5^ Neste estudo, o tempo mediano entre o nascimento e o diagnóstico foi de 22 meses e o tempo mediano entre a primeira consulta e o diagnóstico foi de 2.4 meses.

Dados americanos e europeus reportam idades medianas de diagnóstico de FC de seis meses e cinco meses, respectivamente.^21^ ^and^ 22 O diagnóstico tardio de FC ocorre em países desenvolvidos entre os pacientes com expressão genotípica distinta, doença pulmonar leve e ausência de sintomas gastrointestinais.^23^ Em um país em desenvolvimento como o Brasil, além das razões acima mencionadas, o diagnóstico tardio parece resultar do encaminhamento atrasado de pacientes para um centro de referência, pois a doença não é reconhecida imediatamente.

Em nosso estudo, tempos maiores entre o nascimento e o diagnóstico e entre a primeira consulta e o diagnóstico foram associados com uma melhor correlação A/I; além disso, o início tardio dos sintomas respiratórios foi associado com uma melhor razão dos índices A/I e IMC/I. Essas associações são, provavelmente, devidas à presença de pacientes com doença pulmonar leve e sem sintomas sugestivos de FC e, consequentemente, um menor impacto da doença sobre o crescimento e estado nutricional, pois se sabe que a melhoria da função pulmonar está associada com o aumento do IMC durante a infância.^24^

Houve melhoria do estado nutricional com o aumento dos valores de CVF (%), VEF1 (%) e FEF_25-75%_, conforme analisado pelo índice IMC/I.

De acordo com a literatura, a saúde pulmonar depende do estado nutricional inicial do paciente. Os pacientes que se recuperam de um estado nutricional ruim em até dois anos após o diagnóstico apresentam melhor função pulmonar e menos tosse aos seis anos, o que enfatiza a necessidade de um tratamento abrangente e agressivo, implementado o mais rapidamente possível após o diagnóstico.^25^

As mudanças de peso e altura têm sido reconhecidas como um dos fatores prognósticos de gravidade da doença e menor sobrevida em pacientes com FC. Uma limitação do nosso estudo é que a espirometria foi feita em pacientes com mais de sete anos e não incluiu toda a população de pacientes com FC.

A incidência de baixo peso e altura diminuiu durante o tratamento neste estudo. Na primeira consulta no serviço, 40,4% dos pacientes apresentaram escore Z <-2 de acordo com o índice IMC/I, *versus* 7,7% dos pacientes avaliados antes da implantação da triagem neonatal. Para o índice E/I, 38% dos pacientes tiveram escores -Z <-2 *versus* 7,7%.

A alta porcentagem de pacientes com FC com íleo meconial (23%) poderia justificar a alta porcentagem de pacientes desnutridos em seus primeiros anos de vida. Os pacientes com íleo meconial tratados cirurgicamente nos primeiros dias de vida sofrem efeitos agravantes que podem prejudicar o seu estado nutricional e o estresse cirúrgico pode dificultar a alimentação no pós-operatório durante uma fase importante de crescimento. Mas esses pacientes tiveram uma boa recuperação nutricional, sem comprometer seu estado nutricional e de crescimento. Devido a melhorias nas técnicas cirúrgicas neonatais, melhores cuidados pós-operatórios e suporte nutricional, o estado nutricional e a sobrevida dos pacientes com FC com íleo meconial parecem ser semelhantes aos pacientes com FC sem íleo meconial. A incidência de íleo meconial relatada por Alvarez et al., no mesmo Centro de Referência de FC, foi de 5,8%.^23^ A hipótese associada com a baixa incidência de íleo meconial no último estudo foi que os pacientes com FC morreriam no primeiro ano de vida, antes que o diagnóstico fosse feito.^23^ No presente estudo, foi detectado íleo meconial em 23% dos pacientes, um valor próximo dos 15-20% citados na literatura internacional.^26^ Atualmente, com a triagem neonatal, o manejo do íleo meconial pode ser mais eficaz em pacientes com FC.

A altura é uma característica de crescimento herdada profundamente influenciada pela nutrição e pelas doenças. Os pais dos pacientes com FC apresentaram alturas médias maiores em comparação com a média da população. As alturas médias foram de 1,73 m para os pais e 1,62 m para as mães no presente estudo.

De acordo com o Instituto Brasileiro de Geografia e Estatística (IBGE), a altura média do homem brasileiro era de 1,70 m e das mulheres brasileiras de 1,58 m em 2009.^27^ As contribuições genéticas dos pais para a altura da sua prole são influenciadas pela nutrição e pelas doenças, principalmente as doenças crônicas, como a FC. A altura dos pacientes estudados, considerando-se o alvo parental, era adequada. Isso é inconsistente com os dados obtidos em outros estudos, que relataram uma diminuição da altura média entre crianças com FC em comparação com a altura média parental.^28^ Assim, apesar dos diagnósticos tardios (mediana de 22 meses neste estudo *versus* seis meses em países desenvolvidos), as alturas finais das crianças neste estudo não foram prejudicadas, o que provavelmente reflete o cuidado multidisciplinar adequado oferecido aos pacientes. Esses dados indicam que o estado nutricional dos nossos pacientes pode ter sido semelhante aos obtidos nos principais centros em países desenvolvidos.

Uma criança com classificação de escore Z ≥-2 e <-1, que sugere que a criança se aproxima das categorias de baixo peso ou altura para a sua idade, merece a atenção dos profissionais de saúde e cuidadores. Uma criança com esses valores deve ser considerada como pertencente ao grupo de risco para o controle nutricional. Neste estudo, 30,8% dos pacientes de acordo com o índice A/I e 23% dos pacientes de acordo com o IMC/I estavam dentro desse intervalo de monitoramento de risco nutricional. Vale ressaltar que a maioria dos pacientes tinha altura e peso adequados, de acordo com os índices estudados, mas as variações de peso na FC são muito frequentes, o que requer atenção especial para esses eventos.

Crianças com FC apresentam insuficiência pancreática no momento do nascimento. O baixo peso ao nascer está presente até mesmo na ausência de prematuridade, na medida em que alterações no crescimento fetal devido ao mau funcionamento do pâncreas exócrino reduz a nutrição intrauterina e resulta em menor peso ao nascimento.^29^ Em nosso estudo, 23,5% dos pacientes apresentaram baixo peso ao nascimento e 18,6% eram prematuros. Esses valores excedem aqueles relatados por Festini et al., que avaliaram as características neonatais de crianças com FC na Itália.^30^ Eles demonstraram que os pesos dos recém-nascidos com FC são em média 246 g menores do que os da população sem FC e que crianças com FC têm um maior risco de nascer prematuras, pequenas para a idade gestacional e com baixo peso do que as crianças sem FC.^30^

Os pacientes que são menos frequentemente hospitalizados têm uma relação A/I melhor. Entre os pacientes com FC, a maior necessidade de ingestão de energia está associada com aumento da demanda calórica devido a repetidas infecções e inflamação das vias aéreas, diminuição da ingestão de alimentos durante os episódios de doença respiratória aguda e diminuição da absorção de gordura. Esse déficit de energia provoca ou agrava a desnutrição e é comum em pacientes hospitalizados. Isso leva a retardo do crescimento, diminuição da força muscular, fadiga, infecções respiratórias de repetição e função pulmonar diminuída e reduz, portanto, a sobrevivência de pacientes com FC.

Apesar da alta prevalência de prematuridade, baixo peso ao nascer e colonização de *P. aeruginosa*, os pacientes estudados apresentaram um bom estado nutricional e crescimento durante a infância. Provavelmente o tratamento oferecido em nosso centro de referência está associado a esse resultado. O Centro de Referência oferece consultas com profissionais de saúde de quatro especialidades, pneumologia, gastroenterologia, fisioterapia e nutrição, e o Estado de São Paulo, no Brasil, oferece suplementos alimentares livres de custo quando prescritos por um médico ou nutricionista para pacientes com FC desde 2003. Essa política tem assegurado uma melhor adesão ao tratamento e melhor nutrição. Além disso, uma organização não governamental formada por pais, chamada Fibrocis, tem um trabalho paralelo com visitas domiciliares feitas por assistentes sociais e assistência financeira para transporte de pacientes para as consultas.

O presente estudo é único porque é o primeiro a avaliar como diversas manifestações da FC afetam o crescimento e o estado nutricional de crianças que foram submetidas a tratamento para FC, mas que não foram diagnosticadas ao nascimento pela triagem neonatal no Brasil. No entanto, há várias limitações. Apesar de alguns resultados serem baseados em um formulário preenchido pelos médicos responsáveis por esses pacientes, alguns testes não são feitos rotineiramente devido a restrições de custo e de financiamento do governo. Outra limitação é que os dados foram coletados em um único grande centro no Brasil e nossos resultados podem não refletir o estado nutricional e o crescimento de todas as crianças brasileiras com FC.

Em conclusão, várias manifestações da FC influenciaram o crescimento e o estado nutricional durante o tratamento, mas os pacientes apresentaram um bom estado nutricional, com uma baixa taxa de desnutrição e crescimento adequado. No entanto, muitos deles estavam na faixa de risco nutricional e necessitavam de atenção especial em relação à perda de peso e ao crescimento adequado. O manejo nutricional adequado determina os resultados.

É fundamental que os nutricionistas, pediatras e outros profissionais envolvidos no cuidado de pacientes com FC reconheçam o processo de crescimento dos pacientes desde a primeira consulta para permitir a intervenção precoce no momento do diagnóstico, antes que ocorra desnutrição significativa. A atenção dada na infância pode ter impacto sobre o estado nutricional, o crescimento, a saúde pulmonar e na adesão ao tratamento e pode aumentar a sobrevida e a qualidade de vida dos pacientes com FC.
